# Hospital Costs and Long-term Survival of Patients Enrolled in an Enhanced Recovery Program for Open Liver Resection: Prospective Randomized Controlled Trial

**DOI:** 10.2196/16829

**Published:** 2021-02-01

**Authors:** Chris N Jones, Ben L Morrison, Leigh JS Kelliher, Matthew Dickinson, Michael Scott, Claudia Cecconi Ebm, Nariman Karanjia, Nial Quiney

**Affiliations:** 1 Royal Surrey NHS Foundation Trust Guildford United Kingdom; 2 Humanitas University Milan Italy

**Keywords:** enhanced recovery after surgery, enhanced recovery, liver resection, enhanced recovery program, health economics, survival

## Abstract

**Background:**

The clinical benefits of enhanced recovery programs (ERPs) have been extensively researched, but few studies have evaluated their cost-effectiveness. Our ERP for open liver resection is based closely on the guidelines produced by the Enhanced Recovery After Surgery Society (2016). This study follows on from a previous randomized controlled trial. We also undertook a long-term follow-up of the patients enrolled in the original trial alongside an analysis of the associated health economics.

**Objective:**

We aimed to undertake a health economic and long-term survival analysis as part of a trial investigating the implementation of an ERP for open liver resection.

**Methods:**

The enhanced recovery elements utilized included extra preoperative education, carbohydrate loading, oral nutritional supplements, postresection goal-directed fluid therapy (LiDCOrapid), early mobilization, and physiotherapy (twice a day compared with once per day in the standard care group). A decision-analytic model was used to compare the study endpoints for ERP versus standard care provided to patients undergoing open liver resection. Outcomes obtained included costs per life-years gained. Resource use and costs were estimated from the perspective of the National Health Service of the United Kingdom. A decision tree and Markov model were constructed using results from our earlier trial and augmented by external data from other published clinical trials. Long-term follow-up was also undertaken for up to 5 years after the surgery, and data were analyzed to ascertain if the ERP conferred any benefit on long-term survival.

**Results:**

Patients receiving ERP had an average life expectancy of 6.9 years versus 6.1 years in the standard care group. The overall costs were £9538.279 (£1=US $1.60) for ERP and £14,793.05 for standard treatment. This results in a cost-effectiveness ratio of –£6748.33/QALY. Patients receiving ERP required fewer visits to their general practitioner (*P*=.006) and required lesser help at home with day-to-day activities (*P*=.04) than patients in the standard care group. Survival was significantly improved at 2 years at 91% (42/46) for patients receiving ERP versus 73% (33/45) for the standard care group (*P*=.03). There was no statistically significant difference at 5 years after the surgery.

**Conclusions:**

ERPs for patients undergoing open liver resection can improve their medium-term survival and are cost-effective for both hospital and community settings.

## Introduction

Liver resection is the preferred treatment option for many primary and secondary liver tumors. Despite advances in surgery and anesthesia and a corresponding reduction in mortality, liver resection is still associated with a high rate of postoperative morbidity ranging from 15% to 45% [1,2]. Enhanced recovery programs (ERPs) have been shown to reduce this morbidity as well as hospital length of stay following colorectal surgery [3]. Only a small number of cohort studies have compared an ERP with standard care in patients who had undergone a liver resection [4-8]. Prior to the clinical trial associated with this study [9], only 1 randomized clinical trial had been conducted; however, that study examined the use of laxatives and nutritional supplements within an ERP and did not compare this treatment with that of standard care [10]. Several systematic reviews, including meta-analyses with some overlap between the included studies, have concluded that ERPs can be successfully implemented for liver resection and can reduce the length of stay without affecting morbidity, mortality, or readmission rates [11-13]. None of the other liver resection trials measured any markers of quality of life (QoL) and only 1 included any economic analysis, showing a reduction in the hospital charges associated with ERP, but not reporting community costs [6].

A systematic review of economic evaluations of ERPs for colorectal surgery concluded that the current evidence is limited but tends to support the cost-effectiveness of ERPs; moreover, it acknowledges a need for further well-designed trials incorporating both hospital and community costs [14]. A broader systematic review looking into ERPs for several specialties agreed that the implementation of ERPs was cost-effective but more trials are needed to examine out-of-hospital costs [15]. Again, with regard to colorectal surgery, one trial has shown reduced community cost with improved QoL following the implementation of an ERP. This study included both open and laparoscopic surgeries, with the ERP group having a significantly higher proportion of laparoscopic surgeries; however, the results remain encouraging in suggesting an economic benefit with ERPs [16].

We performed a full economic evaluation alongside a randomized clinical trial [9], which incorporated QoL outcomes. Our timeframe was the first 4 weeks after the surgery based on a previous review that showed no difference in the QoL after that period [17]. We also conducted a separate 5-year follow-up of patients enrolled in the original randomized controlled trial to ascertain any significant difference in the long-term mortality between the ERP and standard care groups. Our intentions in this study were to establish whether any health economic benefit could be achieved, both in the hospital and in the community as a result of the introduction of an ERP for open liver resection and to investigate whether any survival benefit was seen over a 5-year postoperative period.

## Methods

### Study Design and Ethical Approval

The economic study was undertaken alongside an randomized clinical trial conducted between March 2011 and May 2012 at a regional hepatobiliary unit in southern England. The trial was ethically approved by the National Health Service (NHS) Research Ethics Committee and monitored by the Trust Research and Development Department. The trial was registered at controlled-trials.com (ISRCTN03274575).

### Participants and Recruitment

All patients presenting for open liver resection were eligible. Patients were excluded if they underwent an entirely laparoscopic operation, needed a second concomitant procedure (eg, bile duct repair), were found to be inoperable at the time of surgery, or were unable to provide consent. Patients were first approached in the outpatient clinic and given a trial information sheet. A second, more comprehensive, discussion took place in the preassessment unit before trial consent was obtained. Patients were then randomized either to treatment within the ERP or standard care. The randomization sequence of group allocation by means of brown opaque envelopes was generated by an independent statistician.

### Perioperative Care

Patients in both groups underwent a standardized anesthetic and surgical technique with thoracic epidurals for postoperative analgesia. The enhanced recovery elements utilized included extra preoperative education, carbohydrate loading, oral nutritional supplements, postresection goal-directed fluid therapy (LiDCOrapid), early mobilization, and physiotherapy (twice a day compared with once per day in the standard care group). Epidurals were removed on postoperative day 2 in the ERP group and on postoperative days 3-4 in the standard care group. The perioperative care is described in more detail in the associated clinical paper [9].

### Quality-Adjusted Life Expectancy

Data from the original trial were used for the clinical outcomes, including survival and complication rates, utility, and costs during the observation period [9]. Utility values for the postoperative period were taken from international published literature and based on the standardized EQ-5D (EuroQoL-5 dimension, EuroQol Group) questionnaire [18] completed in the preassessment clinic after giving informed consent and on postoperative days 2, 3, 5, 7, 10, 14, and 28. The mean age and gender-specific life expectancy for our study population were extracted from UK mortality tables and adjusted for an increased relative risk of mortality in high-risk surgical survivors [19]. We then assigned a utility value to various stages of the disease process to derive the quality-adjusted life expectancy.

### Resources and Costs

**Figure 1:**
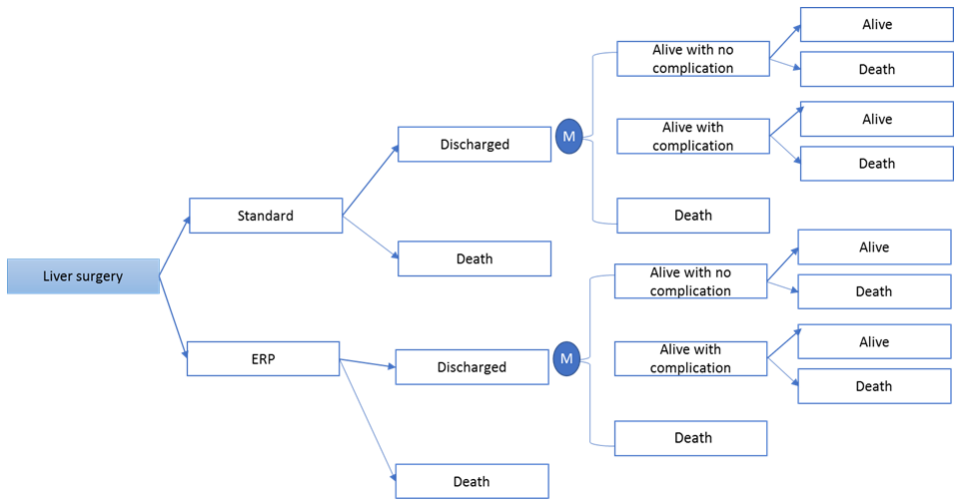
Markov model. After surgery, a patient is either scheduled for standard treatment or an enhanced recovery program. If a patient is discharged, he/she has a certain risk to live with/without complications or die within the subsequent 15 years. ERP: enhanced recovery program.

We explored a number of health economic outcomes—our primary outcome being the incremental cost-effectiveness of implementing an ERP. In order to estimate long-term outcomes, we created a Markov model (Figure 1). This model uses a mathematical algorithm to calculate outcomes based on actual data from the original trial and assumptions based on external evidence [17,18,20,21]. As the model also calculates the lifetime cost-effectiveness, we used the data available on UK health care standard life tables to calculate the life expectancy of our cohort and multiplied with the utility per life year [19]. We ran this simulation model over 10 years, whereby each individual had a possibility to annually transit between the various health states (alive without complications, alive with complications, developed complications, remain in the current state, or die). These data were checked against international literature [17,18]. The calculated time span of our cost-effectiveness model was 10 years, whereby the model was fed with survival trial data for up to 5 years and complemented with a predicted lifetime survival based on the UK life expectancy data. For the purpose of analysis, an episode of care was defined using in-hospital costs (up to discharge) or long-term follow-up costs (10 years).

For the cost-effectiveness analysis, we used trial input data to calculate the mean costs and mean quality-adjusted life expectancy for each treatment arm. These were determined by calculating the expected remaining mean life years per population and multiplying these with the utility of being in these states (mean values). The EQ-5D data were converted into a country-specific utility index value using UK-specific value sets (with values taken from 2014). In a subsequent Monte Carlo simulation, incremental costs and outcomes were computed using repeated random sampling to generate simulated data to use with a mathematical model. The simulation was repeated 10,000 times to calculate the costs, effects, and the incremental cost-effectiveness ratio and its 95% confidence intervals.

**Figure 2:**
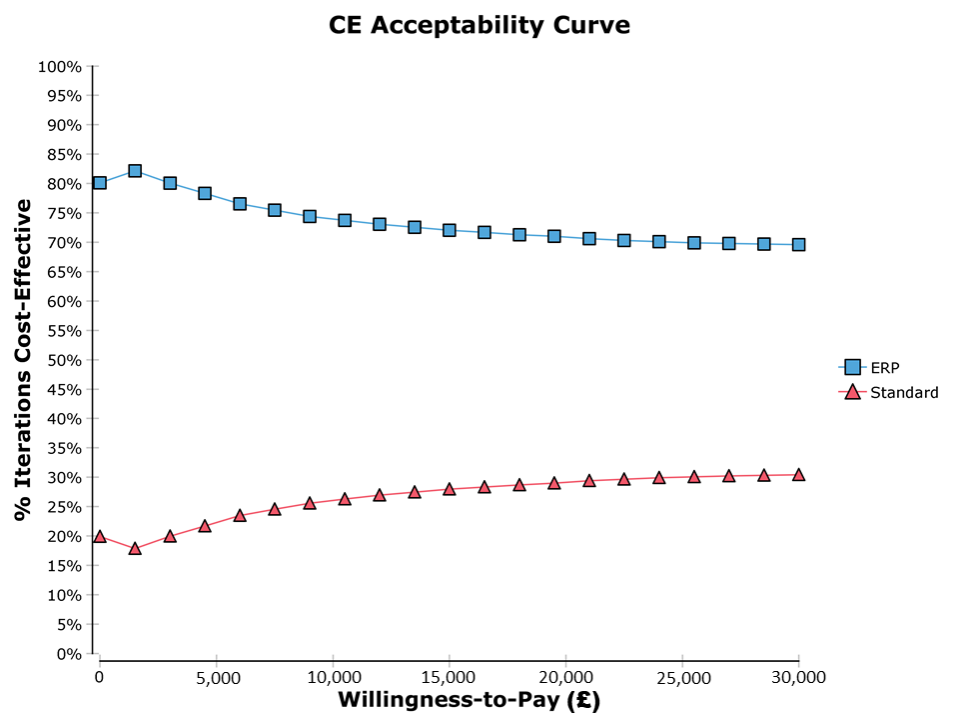
Cost-effectiveness acceptability curve. Throughout a wide range of varying willingness-to-pay, the enhanced recovery program pathway remains the dominant strategy. CE: cost-effectiveness; ERP: enhanced recovery program; £1= US $1.60.

Subsequently, a cost-effectiveness acceptability curve (Figure 2) was plotted to graphically illustrate the outcome with different thresholds for one’s willingness-to-pay for additional benefits gained (eg, willingness-to-pay for gaining an extra life year). The long-term outcomes assessed were costs and quality-adjusted life years (QALYs) gained. Our primary health economic outcome was the incremental cost-effectiveness of the ERP versus that of standard care, which was measured as the ratio of the differences in the costs and differences in the QALYs between the 2 patient groups.

Data on all NHS health care resources used in the treatment for both groups during the first 4 weeks after the surgery, including those relating to the operation, hospital stay, and postdischarge community care, was collected prospectively at the individual patient level. Indirect (societal) costs associated with lost productivity were not calculated due to the short observation period of the study and the technical and conceptual problems associated with assessing them.

The anesthetic and operation techniques were the same for all participants. Operation costs were calculated using the fully absorbed costs obtained locally (see Multimedia Appendix 1). The operation start and finish times were recorded for each patient. Likewise, the anesthetic start and finish times were recorded; thus, anesthetic costs could similarly be calculated using fully absorbed costs obtained from the finance department (£9.16 per minute for anesthesia and £15.70 per minute of theater time, £1=US $1.60). The fully absorbed hospital costs relating to the length of the postoperative hospital stay and use of ward beds, high dependency units, and intensive care units (in days) were obtained locally (intensive care unit £1652.80 per day, high dependency unit £502.08 per day, and ward £151.68 per day). Additional costs for the ERP group included the preoperative carbohydrate drink (Nutricia Clinical Care: 6 cartons £8.40), oral nutritional supplements (Fortisip Compact, Nutricia Clinical Care: contract price £0.14 per bottle), and use of LiDCOrapid (total fixed and variable costs £91.20 per patient). The community health care costs incurred by NHS health care providers in the month after the discharge, including consultations at a general practitioner surgery and home visits by district nurses, were assessed by a questionnaire given to the patient on discharge. They were asked to complete this on postoperative day 14 and repeated on postoperative day 28. Unit costs for community health care resources were obtained from the “Unit Costs of Health and Social Care 2011” compiled by Lesley Curtis (Multimedia Appendix 2) [22]. The costs for patients requiring readmission for overnight stays were included in the hospital costs using the appropriate fully absorbed daily rate.

In order to estimate the long-term costs, we added direct postdischarge health care costs for the follow-up management of high-risk surgical patients and used a 3% discount rate as recommended by the National Institute of Clinical Excellence. In our analysis, we assumed daily costs in hospital to be linear, meaning that the first day has the same monetary value as all subsequent days. This does not reflect the real-world scenario, and therefore, we performed a sensitivity analysis around these values. We ran Monte Carlo simulations to account for variances in model inputs. Mean data were used for the cost-effectiveness acceptability curve to plot the threshold for when a society is unwilling to pay for any additional life gained.

### Five-Year Survival Rates

Patients were followed up at 5 years from the date of their operation by using the NHS Spine data portal. If the patient had died during this period, his or her date of death was recorded and survival after the surgery was calculated. A Kaplan-Meier survival curve was created for each group and statistical significance was calculated at set intervals using chi-square or Fisher exact tests depending upon sample size.

## Results

### Patient Demographics and Care After Surgery

A total of 104 consecutive patients were enrolled in the trial. Thirteen patients were withdrawn after randomization because of changes to their original oncological staging. Ninety-one patients completed the study; 45 received standard care and 46 were treated within the ERP. Patients in the ERP group had significantly higher P-POSSUM (Portsmouth modification of the Physiological and Operative Severity Score for the enUmeration of Mortality and morbidity) scores and a significantly higher proportion of malignant disease (see Table 1). The median anesthetic time was similar in both groups (52 [IQR 45-60] minutes in the ERP group vs 55 [IQR 40-60] minutes for the standard care group, *P*=.64) as was surgical time (189 [IQR 163-236] minutes vs 207 [IQR 150-255] minutes, *P*=.54, respectively). The median total theater cost was, therefore, similar in both groups (£3457 [IQR £3073-£4125] vs £3618 [IQR £2898-£4507], *P*=.55, respectively).

Intensive care unit stays were on average half a day shorter in the ERP group when compared to those of the standard care group; however, this was not significant (1.5 [IQR 1-2] days vs 2 [IQR 1-2] days, *P*=.15). For this level two care, there was a median £827 cost saving in the ERP group; however, this did not reach statistical significance (ERP: £2479 [IQR £1563-£3306] vs standard care: £3306 [IQR £1653-£3306], *P*=.18). High dependency unit care was also 1 day shorter in the ERP group when compared to that in the standard care group but was not statistically significant (1 [IQR 0-2] day vs 2 [IQR 0-3] days, *P*=.08); similarly, there was a median £502 cost saving in the ERP group but this did not reach statistical significance (ERP: £502 [IQR £0-£1004] vs standard care: £1004 [IQR £0-£1506], *P*=.09). There was a 1-day reduction from 3 days to 2 days in normal ward stay in the ERP group versus standard care (*P*<.001). Patients in the ERP group were discharged home, on average, 3 days earlier than the standard care group (median 4 [IQR 3-5] days vs 7 [IQR 6-8] days). Similarly, there was a reduction of £151.68 in the costs between groups that did reach statistical significance (£303 [IQR £0-£341) vs £455 [IQR £303-£758], respectively, *P*<.001).

 

Patients in the ERP group had, on average, 3.9 physiotherapy sessions per hospital episode compared to 4.3 sessions in the standard care group. Overall, the cost per number of bed days between the groups showed an average saving of £654 in favor of the ERP group (*P*=.01). However, when we compared the overall hospital costs, we observed a median cost saving of £864 per patient in favor of the ERP group (ERP: £6826 [IQR £5804-£8124] vs standard care: £7690 [IQR £6880-£9763], *P*=.007). The overall hospital cost for each group showed a £113,476 difference in favor of the ERP group (£344,147 vs £457,623, respectively).

**Table 1:** Patient demographics and operation details (N=91).

Characteristics		Enhanced recovery program group (n=46)	Standard care group (n=45)
Age (years), median (IQR)		64 (27-83)	67 (27-84)
Sex ratio (Male:Female)		31:15	23:22
Body mass index (kg/m^2^), mean (SD)		25.6 (5.0)	26.9 (4.4)
**American Society of Anesthesiologists fitness grade (n)**			
	I	0	2
	II	43	38
	III	3	5
**Diagnosis (n)**			
	Colorectal metastases	35	26
	Other metastases	10	10
	Benign disease	1	9
Neoadjuvant chemotherapy		36	25
**P-POSSUM^a^, mean (SD)**			
	Physiological score	16.4 (3.4)	16.8 (3.6)
	Operative severity score	19.4 (3.7)	17.1 (4.8)
**Operation (n)**			
	Major resection (3 segments)	21	12
	Minor resection	25	33
Specimen weight (g), median (IQR)		373.3 (156.3-780.5)	179.5 (69.6-606.3)
Blood loss (mL), median (IQR)		350 (174-900)	340 (150-645)
Need for blood transfusion (n)		7	3
Death		1	1

^a^P-POSSUM: Portsmouth modification of the Physiological and Operative Severity Score for the enUmeration of Mortality and morbidity.

### Community Care After Discharge

Overall, there was no increase in community or primary care health use after discharge in the ERP group (see Table 2). Despite being discharged, on average, 3 days sooner, fewer patients in the ERP group required visits to their general practitioner, with 15 visits in the ERP group compared with 38 in the standard care group (*P*=.006). Similarly, only 33 patients in the ERP group required visits to a practice nurse compared with 48 in the standard care group during the same period, but again, this did not reach statistical significance (*P*=.12). Significantly fewer patients in the ERP group required help with day-to-day activities at home from friends and family (25 patients vs 33 patients in the standard care group, *P*=.04). There were no significant differences between the groups with regard to outpatient visits (ERP: 5 [11%] vs standard care: 10 [22%], *P*=.17) or emergency department attendances (ERP: 2 [4%] vs standard care: 0 [0%], *P*=.49). Average community care costs based on per patient basis were similar in both groups (median £90 [IQR £21-£156] vs £73 [IQR £47-£203] in the standard care group, *P*=.49). Overall costs of community care showed a £2542 cost saving in favor of the ERP group (£6723 in the ERP group vs £9265 for the standard care group).

**Table 2:** Data of community care and primary care usage by the patients from the day of discharge to postoperative day 28 (N=91).

Postoperative data of patients	Enhanced recovery program group (n=46)	Standard care group (n=45)	*P* value^a^
Visit to general practitioner, n (%)	14 (30)	25 (56)	.01^b^
Total visits to general practitioner (n)	15	38	.006^c^
Home visit from general practitioner, n (%)	0 (0)	1 (2)	.49
General practitioner practice nurse visits, n (%)	20 (43)	28 (62)	.052^b^
Total visits to nurse (n)	33	48	.12^c^
Home visit from nurse, n (%)	17 (37)	17 (38)	.87^b^
Total nurse home visits (n)	42	49	.86
Outpatient visit, n (%)	5 (11)	10 (22)	.16
Emergency department/walk-in center, n (%)	2 (4)	0 (0)	.49
Help from friends and family, n (%)	25 (54)	33 (73)	.04^b^
Total friends and family events (n)	132	188	.02^c^

^a^Statistical significance tested with Fisher exact test. The statistical test chosen was based upon sample size.

^b^Chi-square test.

^c^Mann-Whitney *U* test.

### Incremental Cost-effectiveness Ratio

**Figure 3:**
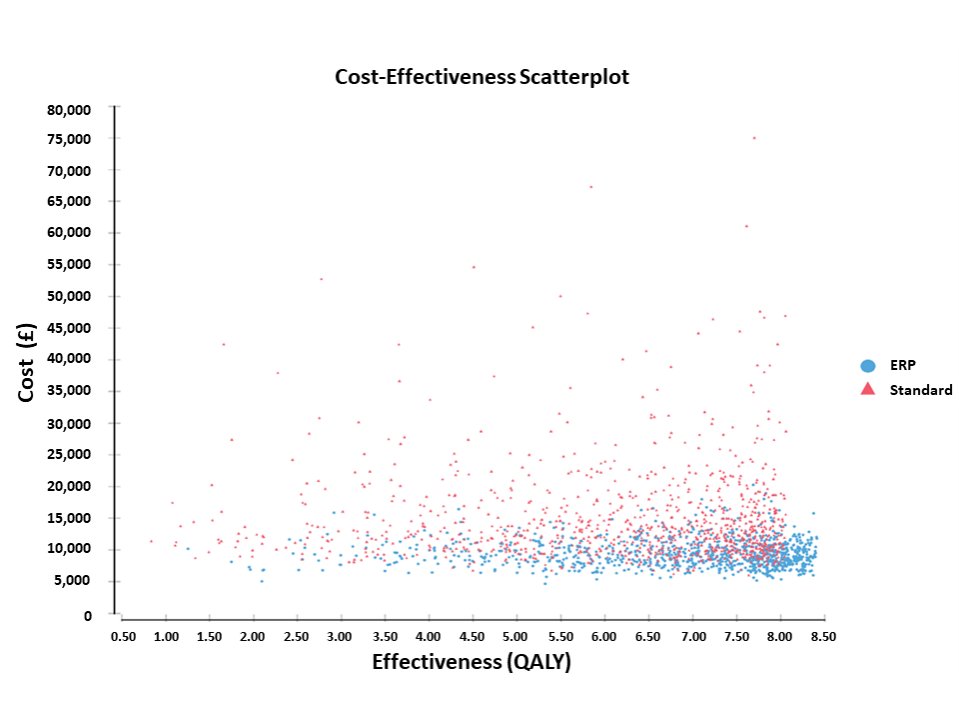
Scatter plot of 10,000 Monte Carlo simulations for the enhanced recovery program demonstrating the costs per quality-adjusted life years for 10,000 independent replications of a patient pathway. ERP: enhanced recovery program; QALY: quality-adjusted life year.

There was a significant difference in the QoL between the 2 groups during the 28 days after surgery, as measured by the multidimensional health value index EQ-5D. The median area under the curve was 37.2 for the ERP group compared with 35.6 for the standard care group (*P*=.002). When this was annualized, it resulted in an overall QALY gain of 0.004 for the ERP group (*P*=.002). Costs were £9538.3 in the ERP versus £14,793.1 in the standard care group, and life expectancy was calculated to be 6.9 years in the ERP group versus 6.1 years in the standard care group. The incremental cost-effectiveness ratio was £–6748.3/QALY gained, meaning that the new pathway is the dominant strategy (more effective and less expensive) and should be recommended to decision makers (Table 3 and Figure 3).

**Table 3:** Results of the incremental cost-effectiveness analysis.

Type of care	Costs (£)^a^	Life years (QALY)^b^	Incremental cost-effectiveness (£/QALY)
Enhanced recovery program	9538.30	6.9	0.0
Standard care	14,793.10	6.1	–6748.30

^a^£1= US $1.60.

^b^QALY: quality-adjusted life year.

### Long-term Mortality

**Figure 4:**
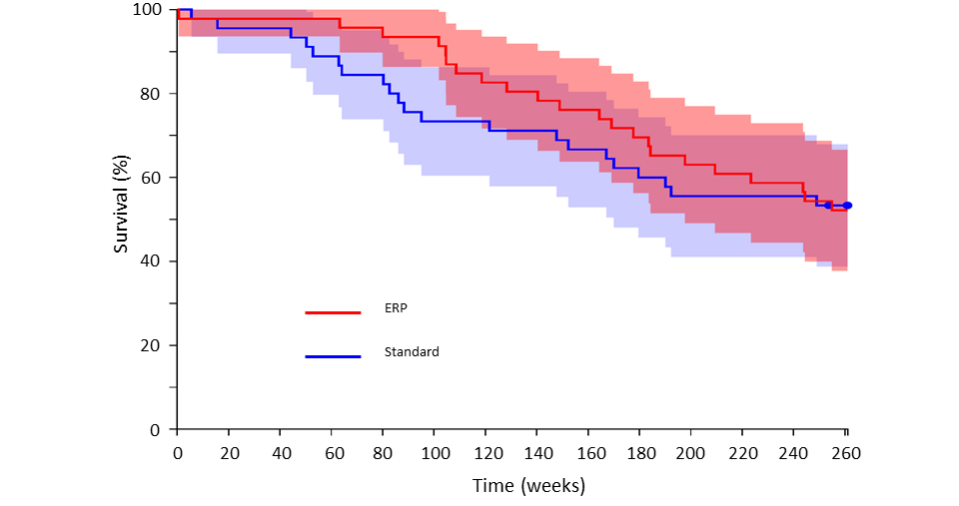
Kaplan-Meier survival curves for all patients. ERP: enhanced recovery program.

**Figure 5:**
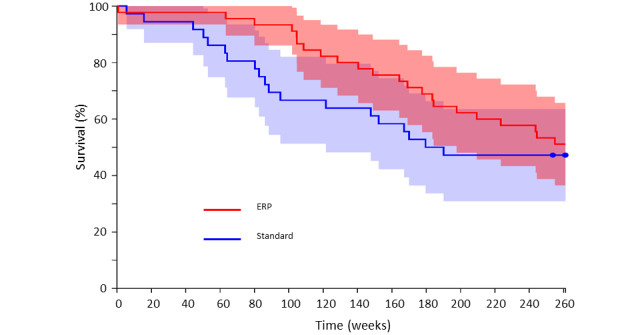
Kaplan-Meier survival curves for patients with malignant disease. ERP: enhanced recovery program.

All patients who underwent surgery were followed up at 5 years and data were analyzed at 1-, 2-, and 5-year intervals. Data are shown in Table 4, Table 5, and Table 6 below. Owing to different sample sizes over the 5-year period, Fisher exact test was used at 1 and 2 years and chi-square test at 5 years to determine statistical significance. Overall mortality was not significantly different between the ERP and standard care groups at 1 and 5 years (survival: 41/45, 91% in standard care group vs 45/46, 98% in ERP group at 1 year, *P*=.20, and 23/45, 51% in standard care group vs 24/46, 52% in ERP group at 5 years, *P*=.92). Patient survival at 2 years was found to be significantly improved (standard care group: 33/45, 73% vs ERP group: 42/46, 91%; *P*=.03), and on subgroup analysis, this difference was more profound in patients with malignant disease (standard care group: 24/36, 67% vs ERP group, 41/45, 91%; *P*=.01), which remained the case when isolating patients with colorectal metastases (standard care group: 18/26, 69% vs ERP group: 32/35, 91%; *P*=.04). Kaplan-Meier survival curves for all patients (Figure 4) and patients with malignant disease (Figure 5) are shown below.

**Table 4:** Long-term survival data of all patients.

Term	Survival of standard care group (n=45), n (%)	Survival of enhanced recovery program group (n=46), n (%)	*P* value
1 year	41 (91)	45 (98)	.20^a^
2 years	33 (73)	42 (91)	.03^a^
5 years	23 (51)	24 (52)	.92^b^

^a^Fisher exact test.

^b^Chi-square test. Choice of statistical test dependent upon the sample size.

**Table 5:** Long-term survival of all patients with malignant disease.

Term	Survival of standard care group (n=36), n (%)	Survival of enhanced recovery program group (n=45), n (%)	*P* value
1 year	32 (89)	44 (98)	.17^a^
2 years	24 (67)	41 (91)	.01^a^
5 years	16 (44)	23 (51)	.55^b^

^a^Fisher exact test.

^b^Chi-square test. Choice of statistical test dependent upon the sample size.

**Table 6:** Long-term survival of patients with colorectal metastases.

Term	Survival of standard care group (n=26), n (%)	Survival of enhanced recovery program group (n=35), n (%)	*P* value
1 year	23 (88)	34 (97)	.30^a^
2 years	18 (69)	32 (91)	.04^a^
5 years	14 (54)	16 (46)	.53^b^

^a^Fisher exact test.

^b^Chi-square test. Choice of statistical test dependent upon the sample size.

## Discussion

Analysis of both in-hospital and community costs showed significant savings for patients in the ERP group, despite inherent cost implications of the pathway itself, alongside reduced mortality at 2 years and the previously demonstrated reduced morbidity and hospital length of stay. Regarding the costs of implementation, the pathway includes a preoperative patient education meeting with a clinical specialist nurse. This was built into their routine preassessment hospital visit, thereby not increasing the burden upon the patient in terms of transport or time off work and not requiring additional nursing staff or appointments. After the surgery, patients in the ERP group received 2 physiotherapy visits per day as opposed to just 1. However, due to the reduced length of hospital stay, this equated to the same number of physiotherapy visits overall. Visits from the acute pain team were reduced due to routine removal of the epidural on postoperative day 2, thereby saving an average of 4 visits.

Importantly, there was no increase in community or primary care costs and having demonstrated an in-hospital cost saving following the implementation of ERP for liver resection, it is reassuring to conclude that costs and burdens have not simply been transferred into the community. In fact, patients in the ERP group required significantly fewer visits to their general practitioner in the first 4 weeks after their discharge, despite being home 3 days earlier. Patients also reported requiring significantly less help at home from friends and family in the first 2 weeks, thus also conferring secondary economic benefits on the part of those who would otherwise have potentially sacrificed time at work themselves.

This is the first time a paper comparing ERP versus standard care for open liver resection has reported on informal caregiver burden. Previous studies have included an economic analysis but did not include any community cost or burden analysis [6,23]. A recent study from Alberta, Canada demonstrated reduced community health service utilization following implementation of an ERP for colorectal surgery [24]. Interestingly, they did not show a significant reduction in primary care visits. Our study does not include any economic benefit from earlier return to work or variation in working days lost due to family or friend assistance, which may result from the ERP. The overall total group costs showed a significant £111,367.60 difference between the groups. However, much of this can be explained by 2 patients in the standard care group who experienced extended hospital stays and contributed over £97,500 of this cost difference. One patient stayed for 39 days with a total hospital cost of £34,623.30. The second patient unfortunately died from liver failure following a prolonged stay in level 2 with hospital costs of £62,921.40. If we exclude these 2 patients from our final analysis, there remains a significant median cost difference of £796.81 per patient between the 2 groups (£7823.88 vs £7027.07, *P*=.02). Note that hospital charges are not the same as direct costs. They serve as a proxy for cost as they are easy to collect but may not accurately resemble true economic cost. Differences between the economic cost, the accounting cost, and the charges to the patient may be different from actual resource use [25]. Being able to demonstrate cost-effectiveness of the ERP should be encouraging to decision makers, considering the implementation of such a program and the financial impact. At the current NHS-recommended threshold of £30,000 per QALY, the probability of the pathway being cost-effective is 73% based upon our analysis.

The ERP group had a significant improvement in survival at 2 years when compared with the standard care group—a finding that is perhaps more noteworthy, given the relatively small number of subjects in the study. The original trial was powered to detect a difference in the length of the hospital stay and thus, it was not anticipated that sufficient patients be recruited to demonstrate any difference in survival with an ERP. Although it is beyond the scope of this analysis to establish why there was improved survival in the ERP group, one possible contributing factor could be the different complication profile between the two groups. In concordance with previous studies, overall complications were significantly reduced in the ERP group [3]. Khuri et al demonstrated a link between the occurrence of postoperative complications and reduced survival time following a major surgery [26]. It follows that if patients experience fewer complications as a result of an ERP, then they may be expected to have improved long-term survival—a hypothesis supported by the findings of a recent systematic review that showed a relationship between postoperative morbidity and worse cancer outcomes following gastrointestinal surgery [27]. In our study, the ERP appears to confer a benefit for roughly the first 2 years following surgery but by 5 years, survival becomes equivalent to those who received standard care. This is contrary to the findings in the study by Khuri et al [26], where the survival benefit was sustained. It could be suggested that the convergence of the curves is a result of the natural history of the overall disease process experienced by patients who require liver resection, but little difference is seen between the groups at 5 years whether patients with benign disease are excluded or not. Only 10 patients of the 91 patients were found to have benign disease of whom 2 died within the 5-year follow-up period. Both were in the standard care group and both died after 2 years (3.7 and 4.8 years). This suggests a 5-year mortality of 20% for patients with benign disease compared to nearer 50% for those with malignant disease although it must be remembered the sample size here is very small. Another factor that may explain the disappearance of the survival benefit at 5 years is the significantly higher P-POSSUM scores in the ERP group, indicating that due to their premorbid health and severity of their surgery, these patients were at higher risk than those in the standard care group.

Overall, this study has shown that an ERP for open liver resection can improve medium-term survival, is cost-effective in both the hospital and community setting, and has the potential to further improve clinical outcomes and incur lower costs to society. In a climate searching for means to increase efficiency and simultaneously improve patient care, enhanced recovery offers the opportunity to achieve both. Initial investment in both money and time will likely be required but the returns have been shown, by this and other studies, to be worth the expense. As more studies are performed, the cost implications are likely to become clearer. For long-term survival rates, more studies would be required to help further establish the ongoing survival benefits, which may be incurred through the implementation of an ERP.

## Appendix

Multimedia Appendix 1 Operation costs.

Multimedia Appendix 2 Unit costs for community health care resources and quality-adjusted life years.

## Abbreviations

ERP: enhanced recovery program
EQ-5D: EuroQoL-5 dimension
NHS: National Health Service
QALY: quality-adjusted life year
QoL: quality of life
