# Supplementary material for: Hospital Costs and Long-term Survival of Patients Enrolled in an Enhanced Recovery Program for Open Liver Resection: Prospective Randomized Controlled Trial
Source: JMIR Perioper Med. 2021 Feb 1;4(1):e16829. doi: 10.2196/16829 (PMC7884210; doi:10.2196/16829)
Supplement: Multimedia Appendix 1 [file periop_v4i1e16829_app1.docx]

**Appendix 1: - Costs**

Resource Costs – Preoperative

| Item | Cost |
| --- | --- |
| preOp Carbohydrate Drink | £8.40 (for six drinks in total)* |
| Fortisips Oral Nutritional Supplement | £2.94 (for 14 drinks in total)** |

*from British National Formulary

** Contract price = 14p per bottle

Resource Costs - Operative

| Item | Cost |
| --- | --- |
| Anaesthetic | £9.16 per minute* |
| Surgery | £15.70 per minute* |
| LiDCOrapid smartcard | £79** |
| LiDCOrapid Monitor | £5930** |
| LiDCOrapid maintenance contract | £577.50 / year (excluding 1^st^ year covered by warranty)** |

* from Royal Surrey County Hospital finance officer – ‘Anaesthetic’ includes costs of anaesthetists, Operating Department Practitioners and consumables, and ‘Surgery’ includes cost of theatre staff and all consumables.

** from LiDCO representative

Resource Costs – Post-operative

| Item | Cost* |
| --- | --- |
| Level 3 Care | £1394.51 / 24 hours |
| Level 2 Care | £1652.80 / 24 hours |
| Level 1 Care | £502.08 / 24 hours |
| Level 0 Care | £151.68 / 24 hours |

*From the critical care group finance officer for the Royal Surrey County Hospital.

Average theatre times (mins) and cost (£) per groups.

*Values presented as median [IQR], with statistical significance tested by Mann-Whitney U*

|  | Standard / Control Group [IQR] | ERP / Treatment Group [IQR] | p-value |
| --- | --- | --- | --- |
| Median anaesthetic time | 55 [40-60] | 52 [45-60] | 0.642 |
| Median anaesthetic cost [IQR] | £503.8  [366.4-549.6] | £476.32  [412.2-549.6] | 0.695 |
| Median surgical time | 207 [150-255] | 189.5 [163-236.25] | 0.541 |
| Median surgical cost [IQR] | £3249.9  [2355-4003.5] | £2975.15  [2559.1-3709.13] | 0.573 |
| Median total theatre time | 250 [207.5-312.5] | 242 [217.5-280] | 0.495 |
| Median total theatre cost [IQR] | £3617.6  [2898.05-4507.3] | £3457.35  [3073.375-4124.57] | 0.546 |

Average median number of days spent, and cost, in each level of care.

*Values presented as median [IQR], significance tested with Mann Whitney U*

|  | Control Group  [IQR] | ERP Group  [IQR] | p-value |
| --- | --- | --- | --- |
| Level 3  Days | 0 [0-0] | 0 [0-0] | 0.564 |
| Level 3  Cost (£) | 0 [0-0] | 0 [0-0] | 0.564 |
| Level 2  Days | 2 [1-2] | 1.5 [1-2] | 0.151 |
| Level 2  Cost (£) | 3305.6  [1652.8-3305.6] | 2479.2  [1652.8-3305.6] | 0.181 |
| Level 1  Days | 2 [0-3] | 1 [0-2] | 0.075 |
| Level 1  Cost (£) | 1004.16  [0-1506.24] | 502.08  [0-1004.16] | 0.085 |
| Level 0  Days | 3 [2-5] | 2 [0-2] | **<0.001** |
| Level 0  Cost (£) | 455.04  [303.36-758.40] | 303.36  [0-341.28] | **<0.001** |

Comparison of overall hospital costs between groups

*Values presented as median [IQR], significance tested with Mann Whitney U*

|  | Control Group  median [IQR] | ERP Group  median [IQR] | p-value |
| --- | --- | --- | --- |
| Overall cost per bed days | £4262.72  [3211.36-5742.88] | £3608.96  [2420.32-4347.68 | **0.012** |
| Average total hospital costs | £7689.84  [6879.87-9762.82] | £6825.74  [5803.82-8124.08] | **0.007** |
| Total group hospital costs | £457,622.90 | £344,147.10 | Difference = £113,475.80 |

Resource Costs – Community

| Item | Cost |
| --- | --- |
| GP consultation | £30 per average 11.7 minute consultation* |
| Home visit from GP | £99 inc travel* |
| Nurse appointment @ GP surgery | £43 per hour of face to face contact est. 30 min consultation = £21.50* |
| Home visit from Nurse | £73/hr inc travel* |
| Hospital out-patient appt | £100* |
| A&E / Walk-in clinic | £54* |
| Day Treatment – (Portal Vein Embolisation) | £931** |
| Social Care | £128 per 4 hour* |
| Medication | Not enough detail was provided in questionnaires to provide costings |
| Help from friends/relatives | No actual cost to NHS |

*From Unit Costs of Health and Social Care 2011- Compiled by Lesley Curtis

** National Schedule of Reference Costs Year : '2009-10'
