# Supplementary material for: Hospital Costs and Long-term Survival of Patients Enrolled in an Enhanced Recovery Program for Open Liver Resection: Prospective Randomized Controlled Trial
Source: JMIR Perioper Med. 2021 Feb 1;4(1):e16829. doi: 10.2196/16829 (PMC7884210; doi:10.2196/16829)
Supplement: Multimedia Appendix 2 [file periop_v4i1e16829_app2.docx]

**Appendix 2 – QALY**

Area-under-the-curve (AUC) values for the EQ5D-HVI scores in the control and ERP groups.

Median [IQR] analysed by Mann-Whitney U test.

|  | Standard Group | ERP Group | p-value |
| --- | --- | --- | --- |
| AUC | 35.6  [33.6-37.2] | 37.3  [35.5-40.7] | **0.002** |

Annualised adjusted measure or QALY compared between groups.

Median [IQR] analysed by Mann-Whitney U test.

|  | Standard Group | ERP Group | p-value |
| --- | --- | --- | --- |
| QALY | 0.0980  [0.0920-0.102] | 0.1020  [0.0987-0.109] | **0.002** |

Total Community costs

|  | Control Group median + [IQR] | ERP Group  median + [IQR] | p-value |
| --- | --- | --- | --- |
| Median cost [IQR] | £73  [47.25-203] | £90.25  [21.50-155.50] | 0.486 |
| Summative cost | £9265 | £6723 | Difference = £2,542 |

Total median costs compared between groups

|  | Control Group  median + [IQR] | ERP Group  median + [IQR] | p-value* |
| --- | --- | --- | --- |
| Median hospital costs | £7689.84  [6879.87-9762.82] | £6825.74  [5803.82-8124.08] | **0.007** |
| Median 28 day care costs [IQR] | £7864.32  [7326.07-10336.24] | £7027.07  [6105.00-8271.35] | **0.011** |
| Total 28 day costs | £466,889.30 | £355,521.70 | Difference = **£111,367.60** |
